# The relation between ApoB/ApoA-1 ratio and the severity of coronary artery disease in patients with acute coronary syndrome

**DOI:** 10.1186/s43044-021-00150-z

**Published:** 2021-03-16

**Authors:** Rehab Ibrahim Yaseen, Mohamed Hesham El-Leboudy, Hend Mohammed El-Deeb

**Affiliations:** 1 Faculty of Medicine, Menoufia University, Abdelrahman Elsharkawey St., Shebin Elkom, Menoufia Egypt; 2 Shebin Elkom Teaching Hospital, Shebin Elkom, Egypt

**Keywords:** ApoB/apoA-1 ratio, Coronary artery disease, Acute coronary syndrome

## Abstract

**Background:**

Apolipoprotein B is considered the primary protein constituent of low-density lipoprotein. LDL contains variable quantities of cholesterol, but each lipoprotein contains a single ApoB protein. Thus, ApoB is a better index for the LDL circulation if compared to LDL cholesterol. On the contrary, apolipoprotein A-1 is a main structural protein of high-density lipoprotein. It has a major role in reversing cholesterol flow and cellular cholesterol homeostasis once detected. The aim of the study is to measure apo B/apo A-1 ratio in patients with acute coronary syndrome and assess its relationship with the severity of CAD.

A total of 90 patients were enrolled in the study and subdivided into 3 groups: 30 patients of STEMI, 30 patients of NSTEMI, and 30 patients presented with unstable angina. Serum levels of apolipoprotein A-1 and apolipoprotein B were properly measured upon admission, and apo B/apo A-1 ratio was calculated.

**Results:**

Both of Apo B and Apo B/Apo A1 ratio correlated significantly with Gensini scores (*P* value <0.001). High Gensini score patients had significantly high Apo B/Apo A1 ratio with the best cutoff value of 0.8 with sensitivity of 90% and specificity of 70%.

**Conclusion:**

Apo B is an independent risk predictor for the severity of CAD in patients with acute coronary syndromes. Moreover, the Apo B/Apo A1 ratio remains highly significant in patients with high Gensini score.

## Background

Acute coronary syndrome refers to a range of different presentations which are ST-segment elevation myocardial infarction (STEMI) and non-ST-segment elevation myocardial infarction (NSTEMI) and unstable angina (UA). Pathologically speaking, ACS is commonly associated with an atherosclerotic plaque rupture that forms thrombus in the infarct-related artery that leads to severe damages [1]. Dyslipidemia represents a metabolic disorder with a continuous rise in cholesterol and triglycerides’ plasmatic concentrations [2]. Additionally, LDL constitutes the highest atherogenic lipoprotein in fasting blood. It plays a major factor responsible for circulating cholesterol into the artery wall [3].

Apolipoprotein B is considered the major protein constituent of low-density lipoprotein. LDL had variable quantities of cholesterol, but each lipoprotein had only one protein which is ApoB; so ApoB is a better predictor of the number of LDL particles than LDL-C [4]. ApoB was considered a more relevant risk predictor of myocardial infarction than LDL cholesterol in the AMORIS study reports which studied the relationship between apolipoproteins, lipids, and myocardial infarction [5]. On the contrary, apolipoprotein A-1 is a main structural protein of high-density lipoprotein. It plays an important role in reversing cholesterol flow and cellular cholesterol homeostasis once detected [6].

Both ApoB/ApoA1 ratios were considered a better risk predictor to acute myocardial infarction than the TC/HDLc ratio [7]. In this regard, the aim of this research was to investigate patients with ACS and study the relation between apo B/apo A-1 ratio and the coronary artery disease severity.

## Methods

This was a cross-sectional observational study that involved 90 subjects who were eligible for coronary angiography.

The patients were categorized into 3 groups according to their ACS presentation. Group I included 30 patients admitted with STEMI, group II included 30 patients with NSTEMI, and group III included 30 patients with unstable angina. The patients were further subdivided according to their Gensini score (GS) into high Gensini score > 47, intermediate Gensini score from 24 to 47, and low Gensini score < 24 [8].

Those with a history of chronic renal failure, sepsis, severe chronic liver disease, previous coronary stenting, and CABG were excluded from the study. Informed written consent was taken from all patients in the study, and the study was approved by the local ethical committee.

Studied patients underwent detailed history taking, full clinical examination, 12-lead ECG, and laboratory investigations in the form of cardiac enzymes (troponin and CK-MB), serum creatinine level, and serum levels of Apo A-1 and Apo B at the time of admission by nephelometric analysis.

### Statistical analysis

Results were statistically analyzed by statistical Package for Social Science (SPSS, version 22). *F* test with pairwise comparison between different groups was used. Chi-squared (*χ*^2^) test was used for comparison regarding qualitative variables. Pearson’s correlation was used to show strength and direction of association between two quantitative variables. ROC (receiver operating characteristic) curves were used to show the connection between clinical sensitivity and specificity for every possible cutoff for a test or a combination of tests. *P* value was considered significant if <0.05.

## Results

In this study, there was not any significant difference between the studied groups as regard age and sex (*P* value > 0.05) (Table 1).

**Table 1:** Comparison between the studied patients as regard age and sex

Demographic data	Number of cases (***n***=90)		
	Low GS <24 (*n*=22)	Intermediate GS (24–47) (*n*=32)	High GS >47 (*n*=36)
**Age (years)**			
Mean ± SD	58.7 ± 11.06	56.00 ± 9.75	56.00 ± 11.16
Min–max	38–72	38–67	39–65
***F*** **(*P*** **value)**		0.51 (0.62)	
**Gender**			
Male	10 (45.5%)	17 (53.1%)	19 (52.8%)
Female	12 (54.5%)	15 (46.9%)	17 (47.2%)
**χ**^**2**^ **(*P*** **value)**		0.49 (0.82)	

*GS* Gensini score

By comparing the number of diabetic patients between the studied groups, it was found that diabetes mellitus was more prevalent significantly in the patients with high Gensini scores and intermediate Gensini scores than those with low Gensini scores (*P* value <0.05). On the contrary, hypertension did not show significant difference in the studied patients (*P* value >0.05) (Table 2).

**Table 2:** Comparison between the studied patients as regard risk factors

Risk factors	Number of cases (***n***=90)		
	Low GS (*n*=22)	Intermediate GS (*n*=32)	High GS (*n*=36)
**Hypertension**	8 (36.4%)	5 (15.6%)	17 (47.2%)
**χ**^**2**^ **(*P*** **value)**		1.42 (0.22)	
DM	7 (31.8%)	21 (65.6%)	25 (69.4%)
**χ**^**2**^ **(*P*** **value)**		6.96 (0.008)*	

*DM* diabetes mellitus, *GS* Gensini score

In our study, we noticed that patients presented with STEMI and NSTEMI had significantly higher levels of Apo B than those with unstable angina (*P* value <0.05). Moreover, Apo B levels in patients presented with NSTEMI exceeded those with STEMI, but it did not reach significant value (*P* value > 0.05).

Surprisingly, there was no statistically significant difference in Apo A1 levels between the studied patients (*P* value > 0.05) (Table 3).

**Table 3:** Laboratory data of the studied groups

Laboratory data	STEMI (***n***=30)	NSTEMI (***n***=30)	Unstable angina (***n***=30)	***F*** test (***p*** value)
**Apo A:**				2.39 (0.09)
Mean ± SD	127.63 ± 14.25	126.27 + 15.23	119.50 + 16.88	
Min –max	93.54–146.77	76.43–162.21	90.13–162.25	
**Apo B:**				17.32 (0.001*) P1=0.25 P2, P3> 0.05*
Mean ± SD	103.13 ± 21.71	111.86 + 21.32	81.94 +14.88	
Min–max	74.10–172.20	80.10–156.50	42.10–130.20	
**Apo B/ApoA ratio**				16.59 (0.001*) P1=0.08 P 2, P3> 0.05*
Mean ± SD	0.81 ± 0.14	0.89 ± 0.15	0.67 ± 0.13	
Min–max	0.59–1.22	0.63–1.16	0.42–0.93	

*STEMI* ST segment elevation myocardial infarction, *NSTEMI* non-ST segment myocardial infarction, *Apo A* apolipoprotein A, *Apo B* apolipoprotein B, *P1* comparing between NSTEMI group and STEMI group, *P2* comparing between NSTEMI and unstable angina, *P3* comparing between STEMI and unstable angina

*Statistically significant (*P* value >0.05)

By comparing Apo B/Apo A1 ratios between the studied groups, we observed that patients presented with STEMI and NSTEMI had significantly higher ratios than those with UA (*P* value <0.05). Moreover, Apo B/Apo A1 ratios in patients presented with NSTEMI were more elevated than those with STEMI, but it did not reach significant value (*P* value >0.05) (Table 3). Additionally, Gensini score was significantly elevated in patients presented with NSTEMI and STEMI than those with UA (*P* value <0.05) (Table 4).

**Table 4:** Gensini score among the studied patients

Gensini score		Low GS <24	Intermediate GS (24–47)	High GS <47
**STEMI** **(*n*=30)**	No.	6	10	14
	%	20%	33.3%	46.7%
**NSTEMI** **(*n*=30)**	No.	4	11	15
	%	13.3%	36.7%	50%
**Unstable angina** **(*n*=30)**	No.	12	11	7
	%	40%	36.7%	23.3%
***X***^**2**^ **(*P*** **value )**		4.49 (0.03*)		

*STEMI* ST segment elevation myocardial infarction, *NSTEMI* non-ST segment myocardial infarction, *GS* Gensini score

The patients with high GS had significantly higher Apo B levels and Apo B/Apo A 1 ratios than those with low GS (*P* value <0.001). Furthermore, the patients with intermediate GS had significantly higher Apo B levels and Apo B/Apo A1 ratios than those with low GS (*P* value <0.001).

**Fig. 1:**
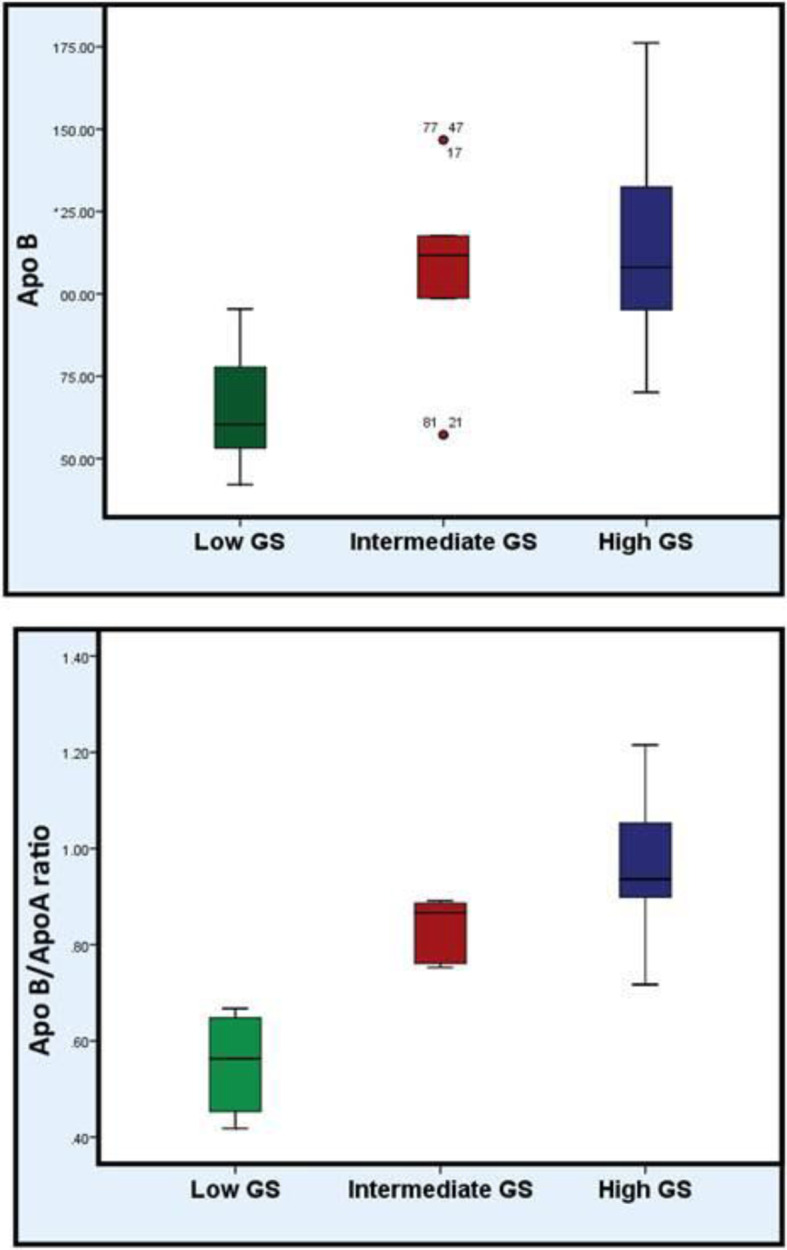
Comparison between the studied patients as regard Apo B and Apo B/ApoA ratio

Surprisingly, only Apo B/Apo A1 ratios were significantly elevated in patients with high GS than those with intermediate GS (*P* value <0.001) but Apo B levels did not show any significant difference between both groups (*P* value >0.05) (Table 5 and Fig. 1).

**Table 5:** Comparison between the studied patients as regard laboratory data

Laboratory data	Number of cases (***n***=90)		
	Low GS (*n*=36)	Intermediate GS (*n*=32)	High GS (*n*=22)
**Apo A:**			
Mean ± SD	118.44 ± 20.41	130.12 ± 43.36	120.67 ± 26.63
Min–Max	93.24–142.96	64.54–192.77	65.78–167.93
***F*** **(*P*** **value)**	0.79 (0.55)		
**Apo B:**			
Mean ± SD	65.77±16.23	106.38 ± 30.20	115.50 ± 29.67
Min–max	42.10–95.40	57.20–146.71	70.10–167.20
***F*** **(*P*** **value)**	18.25 (0.001)*		
**Comparing between groups**	P1 > 0.001*, P2 > 0.001*, P3= 0.59		
**Apo B/Apo A ratio:**			
Mean ± SD	0.59 ± 0.10	0.74 ± 0.06	0.96 ± 0.12
Min–max	0.42–0.69	0.70–0.82	0.79–1.22
***F*** **(*P*** **value)**	77.69 (0.001)*		
**Comparing between groups**	P1 > 0.001*, P2 > 0.001*, P3> 0.001*		

*F* for one-way ANOVA (analysis of variance, Sig. bet. groups were done using post hoc test (Dunn’s multiple comparisons test)), *P1* comparing between low GS and intermediate GS group, *P2* comparing between low GS and high GS group, *P3* comparing between intermediate GS group and high GS group

*Statistically significant (*P* value >0.05)

**Fig. 2:**
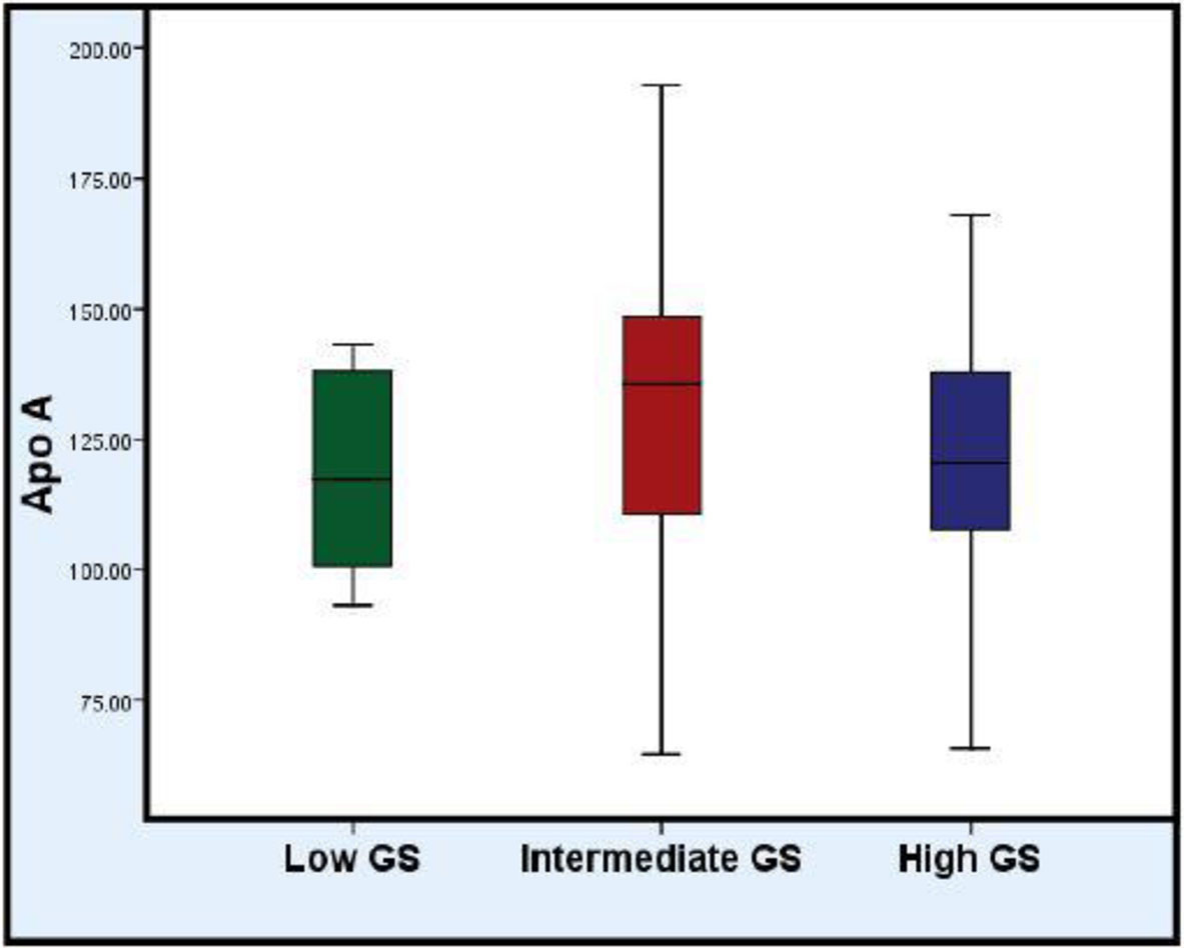
Comparison between the studied patients as regard Apo A

The comparison between the studied groups showed that there was not any statistically significant difference between them regarding Apo A1 levels (*P* value >0.05) (Table 5 and Fig. 2).

**Fig. 3:**
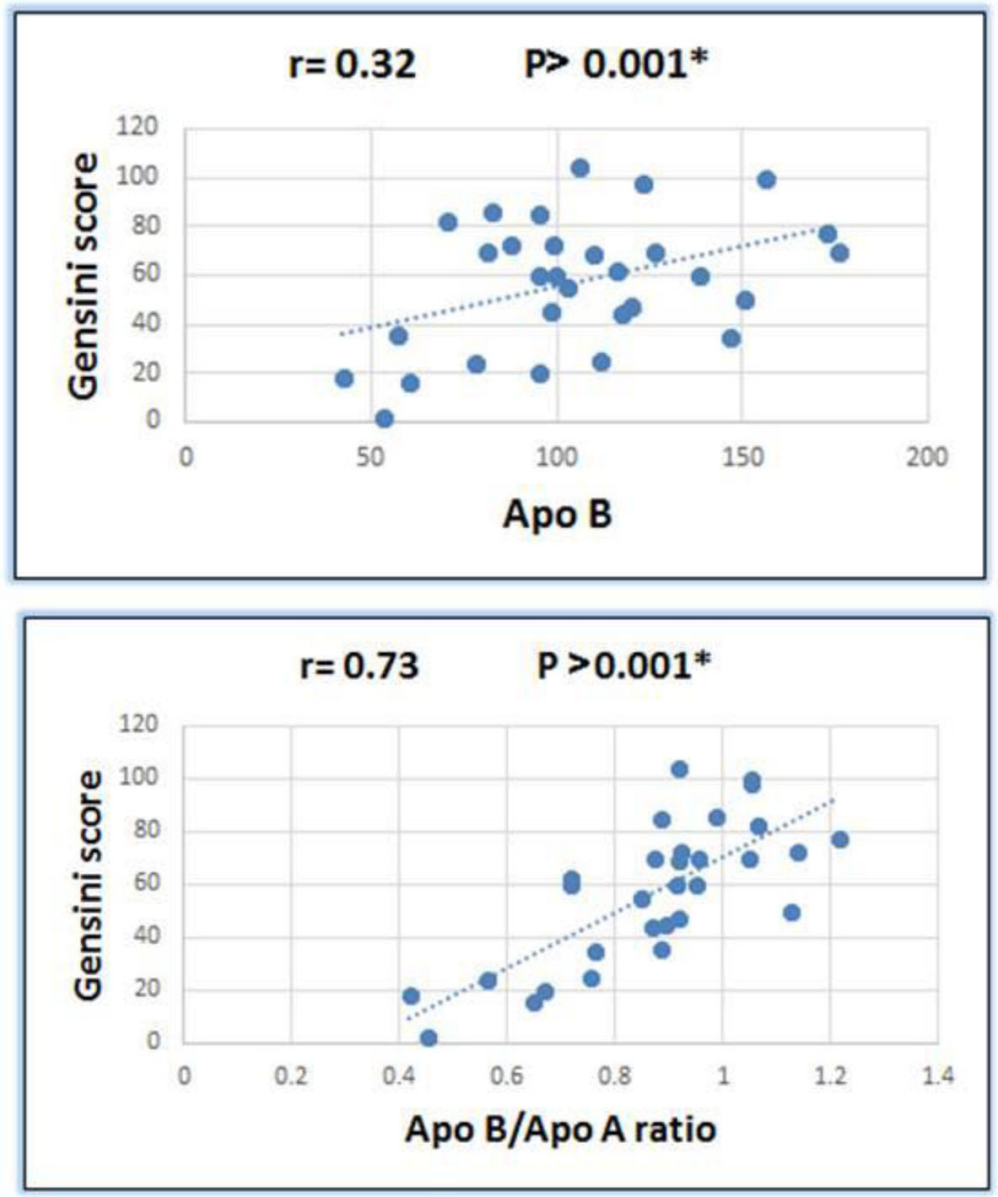
Correlation between both Apo B, Apo B/Apo A ratio, and Gensini score in the studied patients

In our study, Apo B and Gensini scores correlated significantly in a positive manner (*P* value <0.001). Also, there was a significantly positive correlation between Apo B/Apo A1 ratio and Gensini scores (*P* value < 0.001) (Fig. 3). On the contrary, no significant correlation was found between Apo A1 and Gensini scores (*P* value >0.05) (Table 6).

**Table 6:** Correlation between Gensini score and other variables in the studied patients

Variables	Gensini score	
	*r*	*P* value
**Age (years)**	0.06	0.53
**Apo A**	−0.14	0.16
**Apo B**	0.32	> 0.001*
**Apo B/Apo A ratio**	0.73	> 0.001*

*r* correlation coefficient of spearman ‘s correlation

**Fig. 4:**
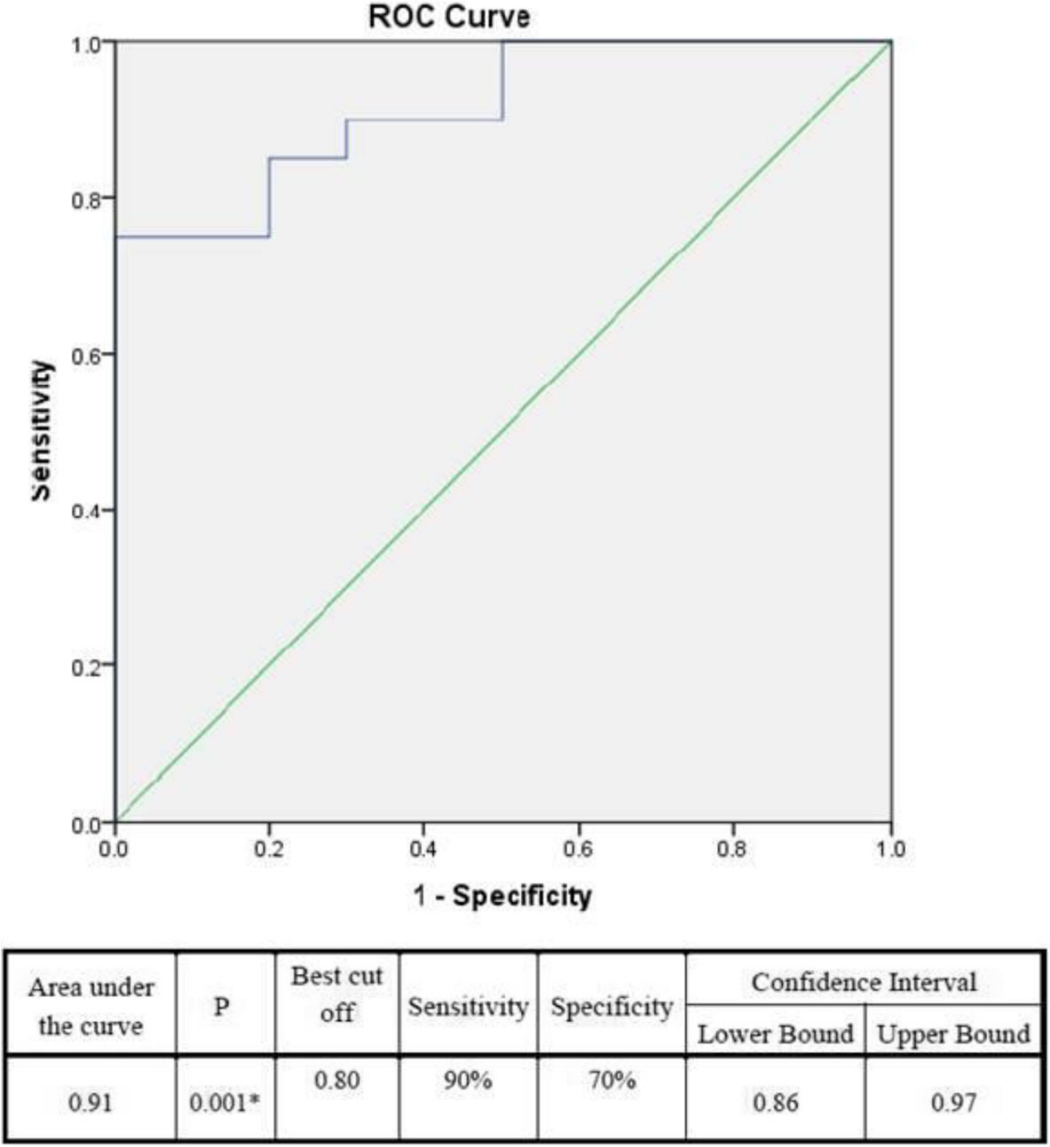
ROC curve of the Apo B/Apo A ratio in patients with high Gensini score

Apo B/Apo A1 ratio was significantly high in patients with high GS with the best cutoff value (0.8) at a 90% sensitivity and a 70% specificity (Fig. 4).

## Discussion

Several epidemiological and clinical studies have constantly and reliably documented that LDL-C constitutes the chief atherogenic lipoprotein for evolving ACS. Substantially, it is viewed as the principal target for lipid-lowering therapies if we targeted to cardiovascular-disease prevention and treatment [9, 10]. Surprisingly, many individuals with ACS may have normal or even low LDL-C concentrations so there was a compelling evidence of developing the prediction of cardiovascular risk by measuring numerous forms of apolipoproteins [11]. ApoB/ApoA1 ratio was recommended as an accountable risk marker of acute myocardial infarction, unlike the TC/HDLc ratio [7].

Our study included 90 patients admitted with ACS. They were divided according to their presentation into 3 groups: STEMI, NSTEMI, and unstable angina. All the patients underwent coronary angiography and were further subclassified according to their Gensini scores into 3 groups: high Gensini score > 47, intermediate Gensini score (24–47), and low Gensini score < 24. Serum Apo B and Apo A1 levels were measured and compared with their Gensini scores.

We found a strong positive correlation of both Apo B and ApoB/ApoA1 in predicting high Gensini scores (scores ≥ 47) in patients who suffered from acute coronary syndromes (*P* value <0.001). Patients with NSTEMI and STEMI had significantly higher Apo B/Apo A 1 ratio and Apo B levels than those with UA (*P* value <0.05). On the contrary, no significant correlation between Apo A and Gensini scores was found (*P* value >0.05). It is known that each particle of the atherogenic lipoproteins like low-density lipoprotein, very-low-density lipoprotein, intermediate-density lipoprotein, and lipoprotein (a) carries a single ApoB100 molecule. Hence, the plasma ApoB100 concentrations notably reflect proatherogenic potentials. On the other hand, ApoA is the key apolipoprotein component of high-density lipoprotein which represents the ApoA serum content whereby antiatherogenic potentials are witnessed. Thus, the ApoB100/ApoA1 ratios represent the balance between the harmful and beneficial potentials. The higher the ApoB100/ApoA1 ratio, the more developed atherogenic potentials we get and/or the less antiatherogenic potentials we attain [12].

Similarly, Tian et al. studied 2256 patients presented with CAD, and they reported a significant association between ApoB/ApoA1 ratios and Gensini scores among these patients [13]. Another study conducted by Song et al. on 792 angiographically defined CAD patients argued that the ApoB/ApoA1 ratios could be a convenient predictor for the coronary stenosis severity in CAD patients [12].

In an evaluation of 170 thousand Swedish subjects, the AMORIS study (Apolipoprotein-related Mortality Risk) showed that Apo B/Apo A-1 ratios were identified as a single variable which was closely associated with attributable risk of fatal MI, predominantly when lipid levels fall within the range of desirable values [5].

Additionally, the INTERHEART study examined about 30 thousand individuals in 52 countries and concluded that the Apo B/Apo A-1 ratios were associated strongly with the prediction of MI than other traditional risk factors as hypertension, smoking, and diabetes regardless of gender, age, and ethnic group [14].

Moreover, Gensini scores presented in this study were significantly elevated in diabetic than non-diabetic patients (*P* value <0.05). These findings come in agreement with a previous study by Du et al. [8] which was carried on 380 diabetic patients, and they concluded that the baseline ApoB/ApoA-1 was typically superior to other lipid and lipoprotein parameters in predicting high GS (scores ≥ 47) in diabetic patients with newly diagnosed CAD. The best cutoff value of ApoB/ApoA-1 in predicting for high GS in DM patients with CAD was 0.72 (with 61.2% sensitivity and 62.1% specificity). In our study, the best cutoff value of ApoB/ApoA-1 was 0.8 (with a sensitivity of 90% and a specificity of 70%).

Similar to our results, Walldius et al. suggested cutoff points for the Apo B/Apo A-1 ratios of 0.8 and stated that the high values could represent an increased hazard of cardiovascular events [15].

Saputri et al. studied 182 patients with ACS and declared that the high Apo B/Apo A-1 ratios ≥ 0.865 resemble an independent predictor of major cardiovascular events with a three-folds risk increase if compared to Apo B/Apo A-1 group ratio < 0.865 during the follow-up [16].

Moreover, Kaneva et al. investigated 157 apparently healthy men with normolipidemia and concluded that the subjects with ApoB/ApoA-1 higher than 0.90 had more atherogenic lipid profile. Thus, the ApoB/ApoA-1 ratio could be classified as a sensitive marker for atherogenic risks [17]. Additionally, Cunanan et al. investigated 95 patients with ACS and revealed the existence of elevated Apo B levels [18].

The Quebec Cardiovascular Study studied 2155 Canadian men to assess superiority of Apo B on other conventional lipid parameters for increased cardiovascular risks. After a 13-year follow-up period, the study remarked the increased Apo B levels as an independent risk factor for ischemic event prediction even with desirable levels of LDL-C [19].

In the study carried out by Hong et al. on 280 patients with NSTEMI who underwent PCI; Apo B and Apo B/Apo A1 ratios were associated with the total occlusion of infarction-related artery in the cases [20]. More recently, Rebecca et al. conducted research on 75 cases with ACS and 69 controls, and they suggested that adding ApoB and ApoB/ApoA1 to the traditional lipid profile would be more helpful in predicting early onset ACS [21].

In a large-scale study, 1414 males and 1436 females with no history of MI were evaluated for 13 years. The results confirmed the strong association between high Apo B levels and increased risk of MI, whereas increased Apo A1 levels were insignificantly associated with low risks of MI. However, the multivariate analysis displayed that the Apo B/Apo A1 ratios were associated strongly with the MI risks, even after age, body mass index, smoking, diabetes mellitus, and hypertension considerations [22].

Hong et al. demonstrated that the ratio of apoB/apoA-1 might confer as the best discriminator for the severity of CAD in diabetic patients; moreover, the higher ratios of apoB/apoA-1 were associated with more severe the CAD would be when assessed with GS [23].

In our study, no significant difference in Apo A1 levels between the studied patients was found (*P* value >0.05). Similarly the Quebec study and the Northwick Park Heart study did not find any significant association between ApoA and CAD risks in multivariable analysis [19]. On the contrary, the AMORIS study concluded that ApoA-1 had an important protecting role against fatal MI. The suggestion retained its close predictive power even if added to a total cholesterol and triglyceride model [5].

One explanation for the lack of statistical significance of Apo-A may be the fact that the present study and several other studies were smaller in sample sizes than AMORIS in which 175,000 participants were included. Thus, such smaller studies might not be eligible enough to express a moderate protective power of ApoA-1 [5].

### Study limitations

There are some limitations of our study that must be addressed: (1) the small number of study population, the full lipid profile was not taken to compare the predictive power of Apo B, and Apo B/Apo A1 ratios with LDL and triglycerides in detection of severe coronary artery disease, we failed to exclude patients who were already on statin therapy before presentation with ACS and lastly there was no follow-up for the patients with higher levels of ApoB and ApoB/ApoA ratios in order to detect early and late cardiovascular events.

## Conclusion

Apo B is an independent risk predictor of severity of CAD in patients presented with ACS. Moreover, the Apo B/Apo A1 ratio remains highly significant in patients with high GS with the best cutoff value 0.8 that may add a benefit in risk prediction.

## Data Availability

All data generated or analyzed during this study are included in this published article.

## Abbreviations

ACS: Acute coronary syndrome
Apo A: Apolipoprotein A
ApoB: Apolipoprotein B
CABG: Coronary artery bypass graft
CAD: Coronary artery disease
CKMB: Creatine kinase-MB
ECG: Electrocardiogram
GS: Gensini score
HDLc: High-density lipoprotein cholesterol
LDL-C: Low-density lipoprotein cholesterol
NSTEMI: Non-ST-segment elevation myocardial infarction
PCI: Percutaneous coronary intervention
ROC: Receiver operating characteristic
STEMI: ST-segment elevation myocardial infarction
TC: Total cholesterol
UA: Unstable angina
